# Supplementary material for: Overview and Evaluation of Existing Guidelines for Rational Antimicrobial Use in Small-Animal Veterinary Practice in Europe
Source: Antibiotics (Basel). 2021 Apr 9;10(4):409. doi: 10.3390/antibiotics10040409 (PMC8069046; doi:10.3390/antibiotics10040409)
Supplement: Supplementary file 1 [file antibiotics-10-00409-s001.zip › antibiotics-1164806-supple/Table S1.docx]

**Supplementary material**

**Table S1: Excluded antimicrobial stewardship guidelines:**

| **Austria** | Leitlinien für den sorgfältigen Umgang mit antibakteriell wirksamen Tierarzneimitteln. Available online : https://www.ris.bka.gv.at/Dokumente/Avn/AVN_20181129_AVN_2018_11a_2/201811a_AntibiotokaLL_Beilage.pdfsig (accessed on 1^st^ March 2021) |
| --- | --- |
| **Germany** | Leitlinien für den sorgfältigen Umgang mit antibakteriell wirksamen Tierarzneimitteln – mit Erläuterungen. Available online : https://www.bundestieraerztekammer.de/tieraerzte/leitlinien/ (accessed on 1^st^ March 2021) |
| **Latvia** | Vadlīnijas antimikrobiālās rezistences attīstības ierobežošanai lauksaimnieciskās ražošanas posmā un veterinārmedicīnas praksē Latvijā. Available online : https://www.bior.lv/sites/default/files/inline-files/Vadlinijas.VPP_.pdf (accessed on 1^st^ March 2021) |
| **Romania** | Ghidul National Privind Utilizarea Prudenta A Antimicrobienelor În Medicina Veterinara. Available online : http://www.ansvsa.ro/download/antimicrobieni/Ghidul-national-privind-utilizarea-prudenta-a-antimicrobienelor-in-medicina-veterinara-actualizat.pdf (accessed on 1^st^ March 2021) |
|  | Norme de conduită în terapia antimicrobiană la câine. Available online : https://cmvro.ro/files/download/antibiorezistenta/Conduita-privind-terapia-antimicrobiana-la-caine.pdf (accessed on 1^st^ March 2021) |
|  | Norme de conduită în terapia antimicrobiană la pisică. Available online : https://cmvro.ro/files/download/antibiorezistenta/Norme-de-conduita-in-terapia-antimicrobiana-pisica_v2.pdf (accessed on 1^st^ March 2021) |
|  | Orientări pentru utilizarea prudentă a substanțelor antimicrobiene în medicina veterinară (2015/C 299/04). Available online : https://cmvro.ro/files/download/noutati/GHID-AMR.pdf (accessed on 1^st^ March 2021) |
| **Serbia** | НАЦИОНАЛНИ ПРОГРАМ ЗА КОНТРОЛУ РЕЗИСТЕНЦИЈЕ БАКТЕРИЈА НА АНТИБИОТИКЕ ЗА ПЕРИОД 2019–2021. Available online : http://www.pravno-informacioni-sistem.rs/SlGlasnikPortal/prilozi/1.html&doctype=reg&abc=cba&eli=true&eliActId=427789&regactid=427789 (accessed on 1^st^ March 2021) |
| **Spain** | Uso responsable de antibióticos en Animales de Compañía. Available online : https://www.vetresponsable.es/vetresponsable/infografias/uso-responsable-de-antibioticos-en-animales-de-compania_4318_299_4620_0_1_in.html (accessed on 1^st^ March 2021) |
| **Sweden** | Antibiotika till hund – behandlingsrekommendation 2016. Available online : https://www.lakemedelsverket.se/48d8cb/globalassets/dokument/behandling-och-forskrivning/behandlingsrekommendationer/behandlingsrekommendation/behandlingsrekommendation-antibiotika-till-hund.pdf (accessed on 1^st^ March 2021) |
